# Segmenting female students' perceptions about Fintech using Explainable AI

**DOI:** 10.3389/frai.2024.1504963

**Published:** 2024-12-12

**Authors:** Christos Adam

**Affiliations:** ^1^Department of Economics, University of Crete, Rethymnon, Greece; ^2^Department of Marine Sciences, University of the Aegean, Mytilene, Greece

**Keywords:** women, Fintech, XAI, clustering, machine learning, SHAP, spectral clustering, classification

## Abstract

The use of Financial Technology (Fintech) has been proposed as a promising way to bridge the gender gap, both financially and socially. However, there is evidence that Fintech is far from achieving this objective, and that women's perceptions of Fintech usages are not clear. Therefore, the main objective of the this study is to segment women's perceptions toward Fintech tools and interpret these segments using machine learning methods. Two primary segments of women were produced, namely a “Fintech-friendly” group and a “Fintech-sceptical” group. The importance and reasonings behind the aforementioned segmentation are then examined. The most prominent factors affecting a woman being in the “Fintech-friendly” group are the perceived benefits of Fintech tools compared to the traditional ones, such as ease of usage, time-space convenience, and its advantageous nature. Finally, for Fintech stakeholders, implications for usability, ease, Fintech education, and tailored experiences may be advantageous approaches.

## 1 Introduction

Financial literacy is an essential skill for skills of our times. According to the OECD (2020) and the research community (Lusardi and Messy, 2023; Panos and Wilson, 2020), financial well-being highly connected with financial literacy in modern societies, being a critical component of economic stability and growth. Many types of financial actions are included, like mortgages (Thorp et al., 2023; Lusardi and Messy, 2023; Choinière-Crèvecoeur and Michaud, 2023), use of cryptocurrency (Alonso et al., 2023; Lusardi and Messy, 2023), and banking (Ferilli et al., 2024). Moreover, financial education is a key step for accomplishing financial inclusion, and thus improving wealth and development worldwide (Khan et al., 2022). However, high financial knowledge percentages are confirmed in many countries (Lusardi and Messy, 2023), touching even the two thirds of the worldwide population (Levantesi and Zacchia, 2021; Lusardi, 2019; Lusardi and Mitchell, 2011). While traditional statistical tools are used by the majority of such studies, the use of machine learning has been introduced very recently in the field (Levantesi and Zacchia, 2021), creating opportunities for relevant applications.

At the same time, it is also well known that one of the most significant sustainable objectives of our age is financial inclusion of women (OECD, 2023) and a means that is supported for bridging this gap is the use of Financial Technology (Fintech) ^1^ (Thylin and Duarte, 2019; Demirguc-Kunt et al., 2018, 2021). For instance, more and more employers and leaders of Fintech companies are women (Loko and Yang, 2022). Despite these, the gender gap is still very large. The root of this issue is the gender discrimination of the current structure of both digital and traditional financial systems, and therefore a more feminist approach is required (Henshaw, 2023). For instance, financial, technological, and entrepreneurial gender inequalities are faced by women attempting to enter the Fintech business environment (Fox-Robertson and Wójcik, 2024). Besides, financial improvement of women through the use of Fintech is hardened in some countries, because of gender discrimination (Esmaeilpour Moghadam and Karami, 2023). In addition, socio-economic background, and regional factors are important for financial and digital literacy of the women (Khera et al., 2022). Therefore, in order to combat against these gender inequalities in financial and social levels, additional research into the perceptions of women toward Fintech tools is essential.

Studying perceptions of women toward Fintech usage has a galloping increase in recent years, despite being an open issue over the last decades (Gefen and Straub, 1997). According to studies, women are inexperienced and uneducated even in traditional means of financial use (Thylin and Duarte, 2019; Demirguc-Kunt et al., 2018, 2021). They literacy gaps about Fintech and their investments, like cryptocurrencies, are seen as risky (Alonso et al., 2023). Despite the decreasing usage gap between men and women, like the one in digital payments, is decreasing but still not eliminated (Demirguc-Kunt et al., 2018, 2021; Galpaya and Zainudeen, 2022). The great interest shown about the psychological and societal factors of this gender gap in the use of Fintech by women is shown mainly with case studies in the existing literature.

Nyhus et al. explored the impact of gender, the financial subjectivity compared to the social opinion, and the Big Five personality traits were explored by Nyhus et al. (2024). In precision, two surveys were conducted, with 126 Scandinavians at the first one and 1,741 Norwegians at the second one. The lower percentage of women's willingness to invest in cryptocurrencies was observed, accompanied by a higher level of congeniality, diligence, closeness, and higher conformity to the social norms, including the assumption of financial self-efficacy.

Reasons for differences between men and women in the approval and use of the decentralized finance were studied by Alonso et al. (2023). Demographic and personal preferences information was extracted according to the Theory of Planned Behavior (TPB) model by surveying 326 Spain inhabitants (about equal gender percentages) using descriptive statistics. According to the authors, the female population is blocked from using of cryptocurrencies because of their inexperience on performing traditional forms of investment and their limited information about modern and traditional financial and digital terms, like cryptocurrency, blockchain, and the notion of exchange, as well as their insecurity and conservatism against cryptocurrency investing. Compared to men, female participants who are negative about or non-users of cryptocurrencies are trustful with conventional means of transaction and are not influenced by digital platforms and their leaders and are not apprehensive about conforming their behavior. Furthermore, women's entrance is not obstructed by their levels of earnings or computer illiteracy.

Participation of women in Fintech leadership was explored by Khera et al. (2022). In particular, using a sample of global company data covering 97 countries and individual data covering 83, the impact of female chiefs on company success in Fintech and the factors influencing the consumption of the digitized technology across countries can be examined. In business, the coexistence of both genders in companies' leadership team for higher earnings and sponsorship was concluded. On an individual level, the inclusion of women in Fintech has a positive relationship with Fintech education, without excluding the effect of societal and ethnic drivers.

Examination of the modernization through the usage of Fintech at the era after the COVID-19 widespread is performed by Igamo et al. (2024). In further detail, the Technology Acceptance Model (TAM) was extended and tested by the impact of governmental aid, Fintech education, existing conditions after the COVID-19 period, and countryside vs. city inhabitants. TAM is a model that interprets the acceptance and usage of technology. There were 403 female participants from Indonesia were surveyed, and these hypotheses were empirically tested by deploying Partial Least Square Structural Equation Models (PLS SEM). Based on the results, the value of the circumstances had the most influence on the usage behavior in the reality after COVID-19. The viewpoint had the biggest impact on explaining goals of individuals behavior and public assistance. Fintech education had high influence in moderating the link between acting goal and usage, with differences between countryside and city population. The necessity of an alternative authority approach among countryside and city Indonesian females in Fintech acquisition is underlined.

Segmenting consumer markets by demographic attributes is a usual strategy (Jaiswal et al., 2023). A well-known theoretical basis in segmenting consumer perceptions and usage is the Unified Theory of Acceptance & Use of Technology (UTAUT) (Venkatesh et al., 2012), which aims to capture behavioral intention, and usage toward a product. One of the multiple variants of this theory was proposed by Jaiswal et al. (2023), wherein the characterization of perceived benefits. Behavior and socio-demographic factors are key factors for segmenting intentions and behavior of consumers toward Fintech. Such an attempt was done by Jaiswal et al. (2023), where responses of 550 people from India about their cashless payment behavior and their personal characteristics such as factor, cluster, discriminant, and Chi-squared analyses were performed. These respondents were grouped into three clusters, namely those who are doubtful about Fintech, those who have preferences about more traditional financial means, and those who are positive about Fintech adoption, even though no gender dependence was revealed between these three groups.

One of the major issues in the Fintech inclusion of the women is sourced from their technology exclusion. For instance, the technology exclusion of the women in Global and Asia South, as well as case studies of India and Sri Lanka were investigated by Galpaya and Zainudeen (2022). In this research, high gender differences in mobile, and internet usage were evidenced. In the two aforementioned case studies, females are blocked from technology utilization because they have limited dexterity levels and knowledge in Fintech capabilities and chances, like digitally self-employed jobs and putting goods or services for sale online.

Therefore, even though gender is an insignificant factor for Fintech usage in some studies (Jaiswal et al., 2023; Ferilli et al., 2024), for the majority of the literature, the positive association between Fintech literature and its usage is observed. Fintech is viewed as a tool for achieving the digital financial tools inclusion of women in many ways. For example, Fintech and the worldwide adoption of the smart-phones are viewed as an opportunity to bring women closer to cryptocurrency (Henshaw, 2023), blockchain (Di Vaio et al., 2023; Thylin and Duarte, 2019), digital bank accounts and payments (Demirguc-Kunt et al., 2018, 2021), financial assets and their transferability, and entrepreneurship (Thylin and Duarte, 2019; Khera et al., 2022). However, both traditional and digital financial education are required for achieving such results (Alonso et al., 2023; Di Vaio et al., 2023). Entering in Fintech usage without first acquiring the proper financial and digital literacy levels and skills are considered as a dangerous and risky practice, despite the gender, for reasons like their complexity (Giudici, 2018). Some different risks in technologies, such as blockchain, that do not relate necessarily to the financial sphere; hence, probably, only digital literacy is needed (Thylin and Duarte, 2019; Ko and Verity, 2016). There are cases where even financially literate individuals might be driven into risky financial actions, but financially literate ones are still in danger (Kawamura et al., 2021). In conclusion, the field about the willingness of the women to use Fintech and the relevant driving factors is obscure and therefore there is an essence for additional research applications concentrated on the intentions and perceptions of the women regarding Fintech. Moreover, work done until now is focused on the utilization of traditional statistical models, and therefore the use of machine learning applications and their interpreting methods in this field are scarce and therefore both scientific community and literature would be benefited from such additions.

In this paper, the perceptions of the women based on their benefits, risks and future usage of Fintech are clustered by using machine learning methods. Thereafter, factors associated with the classification of women to each cluster are examined. To the best of our knowledge, this is the first study using Explainable AI (XAI) in clustering women's perceptions toward Fintech use and so existing literature will be enriched by such insights on the issue. The technical characteristics of the current paper are presented in MATERIALS AND METHODS, the result of their application which are shown in RESULTS and finally in DISCUSSION are illustrated some concluding remarks and implications derived from this study.

## 2 Materials and methods

The dataset from the survey conducted by Al Azizah et al. (2022) was utilized. Constructing a reliable dataset comprising university students' perceptions of the benefits and risks associated with Fintech adoption, and how these components affect their readiness for Fintech, was the objective of this survey. Consisting of 436 Indonesian students, information about their gender, monthly income, semester, field of study, and graduation is contained in this dataset. Groups of Likert-type questions regarding perceived and economic benefits, facilitations, ease of transactions, willingness for future use, and financial, legal, operational, and security risks related to Fintech usage are included. After collecting and preprocessing these survey data and describing their statistics, PLS SEM were employed by Al Azizah et al. (2022). Only female participants were examined in the current study. This step was taken to focus the analysis more on segmenting female students' perceptions of Fintech, resulting in 315 respondents. To provide as much information as possible about the correspondents, all remaining 36 categorical and ordinal features were included in this subset.

From a technical point, the preferred clustering algorithm was Spectral clustering (Shi and Malik, 2000; Ng et al., 2001; Meilă and Shi, 2001; Ding, 2004). In this algorithm, a similarity matrix is constructed by finding nearest neighborhoods of a dissimilarity matrix. Then, a Laplacian matrix is computed by the graph created from a similarity matrix, and the largest *k* eigenvectors of this matrix are finally used as features to a clustering algorithm, like k-means or Gaussian Mixture Models (GMM) (Zelnik-Manor and Perona, 2005). This method is suitable for clustering a small number of instances with significant dimensions, provided that the eigenvectors are usually less than the initial number of features. For identifying the optimal number of clusters, the eigengap heuristic was utilized (Von Luxburg, 2007). After, a clustering algorithm at the selected eigenvectors was applied, excluding the first non-informative eigenvector. However, for the specific case that only *k* = 1 informative eigenvector is used, named also Fiedler vector (Fiedler, 1973) partitioned the adjacency graph of the similarity matrix into two graphs, based on the signs of values of the Fiedler vector, instead of employing k-means or GMM.

The Gower dissimilarity matrix (Gower, 1971; Kaufman and Rousseeuw, 1990; Struyf et al., 1997) was calculated. Capability of computing dissimilarity matrix for dataset containing only categorical and ordinal features was the reason it was selected. However, in order to transform it into a similarity matrix, mutual nearest neighborhoods were computed using this dissimilarity matrix. It should be noted that mutually nearest neighborhoods do not have a specific rule of thumb for the optimal number of neighborhoods and it can be larger than the usual nearest neighborhoods method (Von Luxburg, 2007). This similarity matrix was used for selecting the most relevant features for clustering by utilizing the Unsupervised Spectral Feature Selection Method for Mixed Data (USFSM) (Solorio-Fernández et al., 2017). According to this filter feature selection method, spectral clustering is performed by removing one feature of a dataset at a time and computing the relevance of that removed feature, based on its impact on first *k* eigengaps. Irrelevance of feature is indicated by negative values of relevance measure ϕ and relevance of feature by a positive one. A huge benefit of this variant method is that it can be used for any feature type. Then, the spectral clustering algorithm was performed for these features. Following the methodology of Louhichi et al. (2023), a classification algorithm for predicting clusters from spectral clustering, namely Random Forest, for the given data was employed. Hyperparameter tuning for the Random Forest model was done by the Bayesian Optimization Gaussian Process Upper Confidence Bound (GP-UCB) method (Srinivas et al., 2012). Then, SHapley Additive exPlanations (SHAP) values for this classifier were estimated. SHAP (SHapley Additive exPlanations) is a way for unveiling the relationship of features with a target in a game-theoretical way, by calculating the total payoff of a cooperative game. This is a usual method for interpreting machine learning black-box models. Here the approximate method of Štrumbelj and Kononenko (2014) was utilized, according to the realization of Greenwell (2024).

## 3 Results

**Figure 1:**
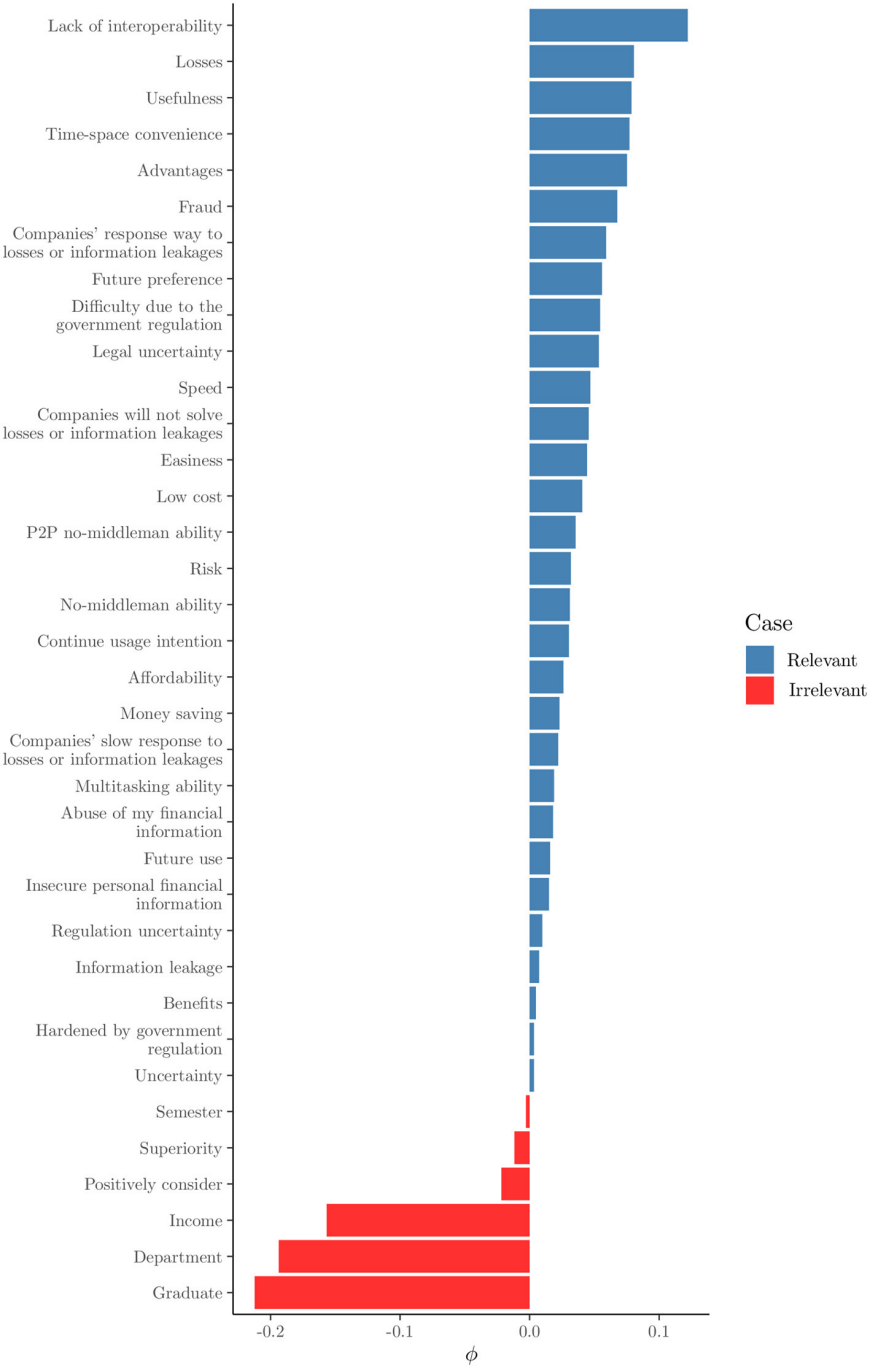
USFSM relevance.

**Figure 2:**
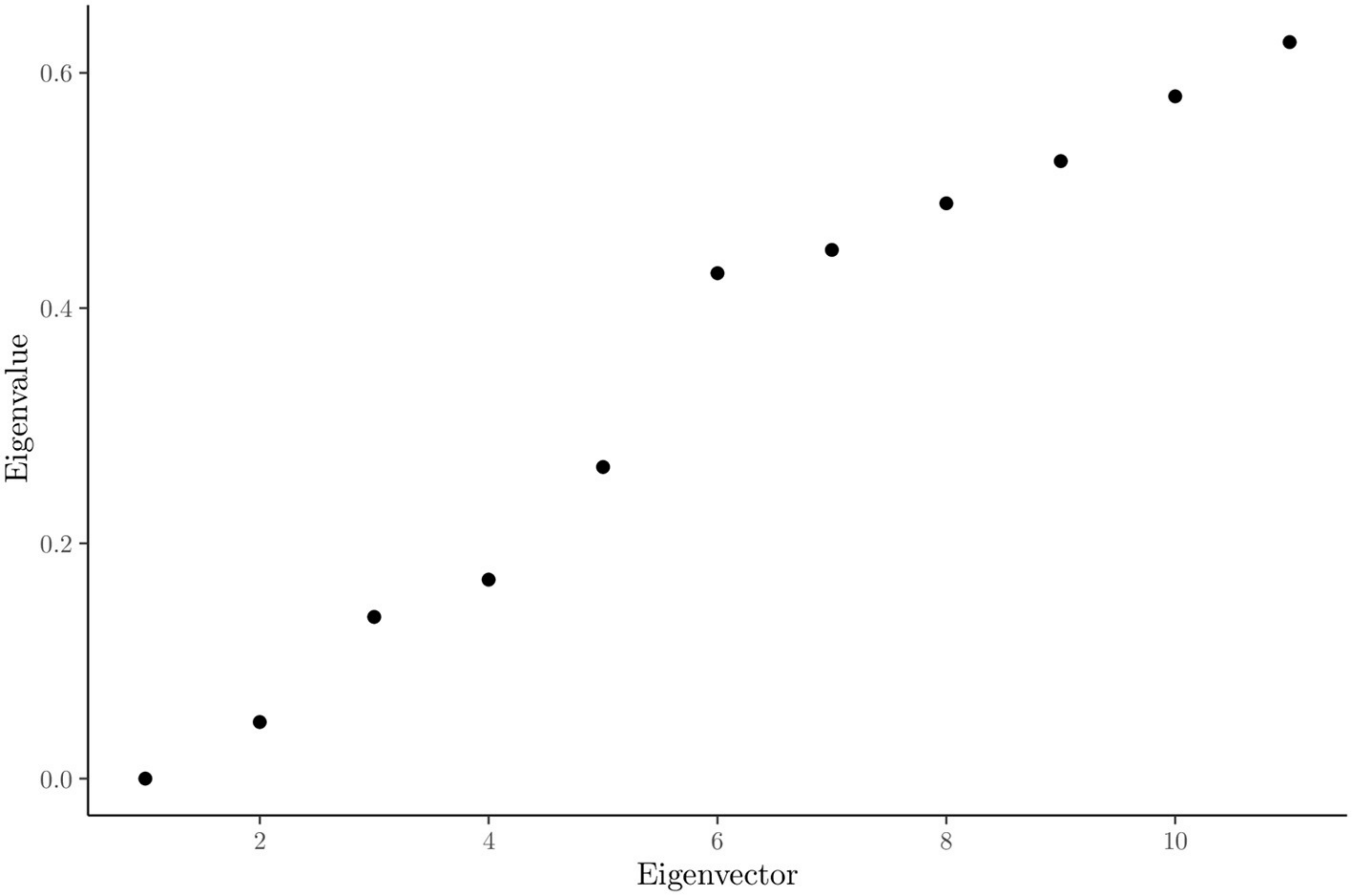
Eigengap for 11 smallest eigenvalues.

**Figure 3:**
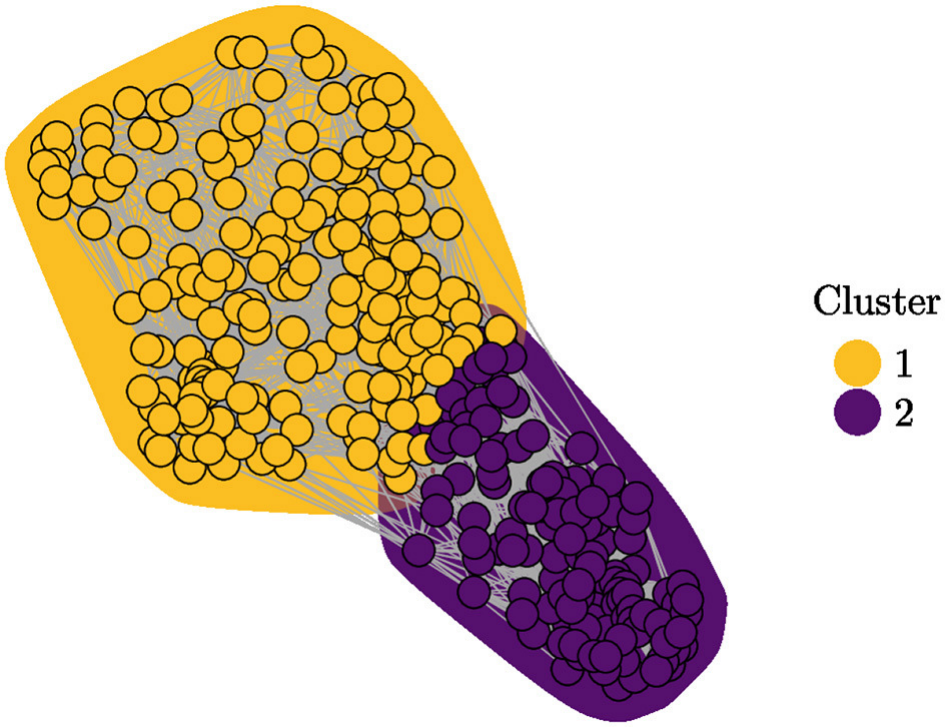
Graph plot of similarity matrix for two clusters.

**Figure 4:**
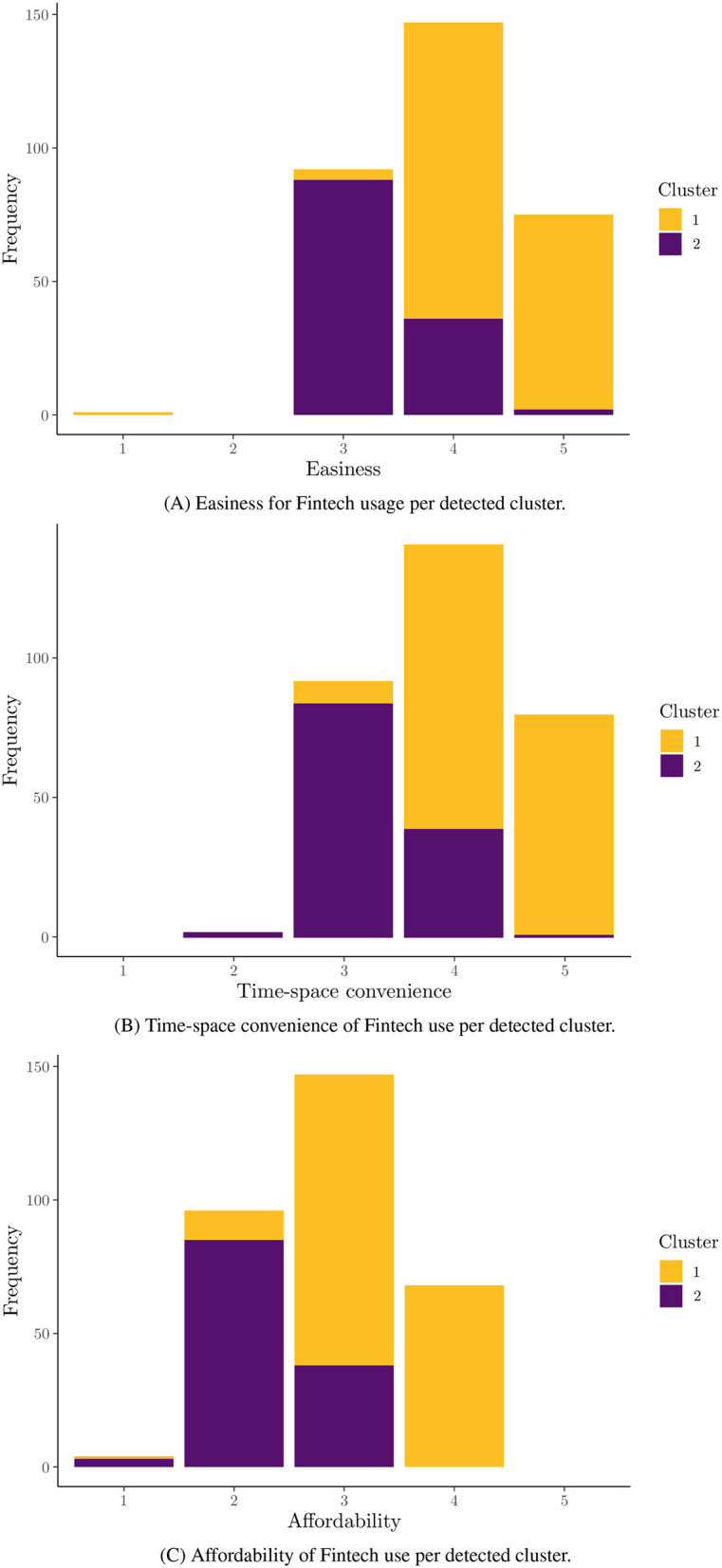
Barplots representing some indicative feature distributions per cluster. **(A)** Easiness for Fintech usage per detected cluster. **(B)** Time-space convenience of Fintech use per detected cluster. **(C)** Affordability of Fintech use per detected cluster.

In Table 1, the abbreviated versions of the original questions asked of the students are presented for practical reasons. In Figure 1, the relevance values for each feature after applying USFSM are presented in descending order. According to this, six irrelevant features are removed, in which all demographic information of the participants is contained. In Figure 2, the eigenvalues for each of the first 11 eigenvectors are shown, including the first zero non-informative one. According to the eigengap heuristic, only the second eigenvector is selected, because the relative gap is larger in comparison to the next one, and it is the first eigengap of near zero eigenvalues (Von Luxburg, 2007). Therefore, only two clusters are present in the examined dataset. By applying Fiedler vector partitioning, these two clusters were identified. In Figure 3, these two partitions are depicted in the graph of the similarity matrix, where each cluster is quite dense within each one cluster, and there is very little overlap among two, but connections among them are visible. So, 189 and 126 instances were contained in Cluster 1 and Cluster 2, respectively. Moreover, descriptive statistics for three indicative predictors at the studied dataset are depicted in Figure 4, namely ease, time-space convenience, and affordability. The preference of instances of Cluster 1 has higher feature values than Cluster 2 is observed in all of them. Thereafter, Random Forest for this binary classification was trained.

**Table 1:** Renaming survey questions into abbreviated feature names.

**No**.	**Question**	**Feature name**
1	Department	Department
2	Semester	Semester
3	Income/Money Every Month (IDR)	Income
4	Graduate	Graduate
5	Using Fintech has many advantages	Advantages
6	Using Fintech is useful for me	Usefulness
7	Using Fintech yields a more superior outcome quality than traditional financial services	Superiority
8	Using Fintech is associated with a high level of risk	Risk
9	There is a high level of uncertainty using Fintech	Uncertainty
10	Overall, I think that there is little benefit to use Fintech compared to traditional financial services	Benefits
11	Using Fintech is cheaper than using traditional financial services	Affordability
12	I can save money when I use Fintech	Money saving
13	I can use various financial services with a low cost when I use Fintech	Low cost
14	I can control my money without the middleman when I use Fintech	No-middleman ability
15	I can use various financial services at the same time (e.g., one stop processing) when I use Fintech	Multitasking ability
16	I can have the peer-to-peer transactions between providers and users without middle man when I use Fintech	P2P no-middleman ability
17	I can use financial services very quickly when I use Fintech	Speed
18	I can use financial services anytime anywhere when I use Fintech	Time-space convenience
19	I can use financial services easily when I use Fintech	Easiness
20	Financial losses are likely when I use Fintech	Losses
21	Financial fraud or payment frauds are likely when I use Fintech	Fraud
22	Financial losses due to the lack of the interoperability with other services are likely when I use Fintech	Lack of interoperability
23	My use of Fintech is uncertain due to many regulations	Regulation uncertainty
24	It is not easy to use Fintech due to the government regulation	Hardned by government regulation
25	There is a legal uncertainty for Fintech users	Legal uncertainty
26	It is difficult to use various Fintech applications due to the government regulation	Difficulty due to the government regulation
27	I worry about the abuse of my financial information (e.g., transaction and private information) when I use Fintech	Abuse of my financial information
28	My financial information is not secure when I use Fintech	Information leakage
29	I worry that someone can access my financial information when I use Fintech	Someone can access my financial information
30	Fintech companies are not willing to solve the issues when financial losses or financial information leakages occur	Companies will not solve losses or information leakages
31	The organizational responses of Fintech companies are too slow when financial losses or financial information leakages occur	Companies' slow response to losses or information leakages
32	I worry about the way Fintech companies respond to financial losses or financial information leakages	Companies' response to losses or information leakages
33	I would positively consider Fintech in my choice set	Positively consider
34	I would prefer Fintech	Future preference
35	I intend to continue to use Fintech	Continue usage intention
36	I will use Fintech in the future	Future use

**Figure 5:**
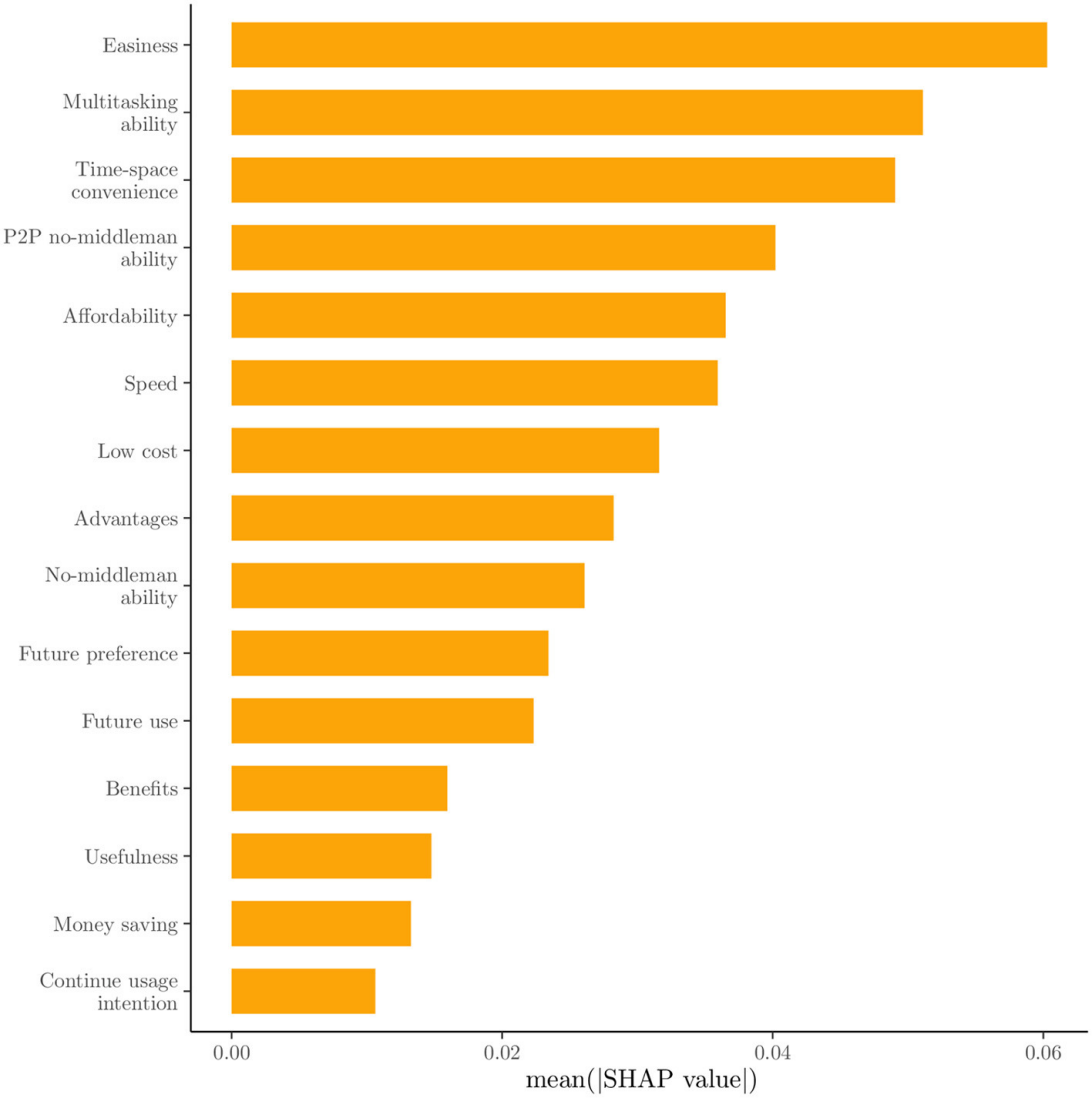
SHAP value feature importance.

**Figure 6:**
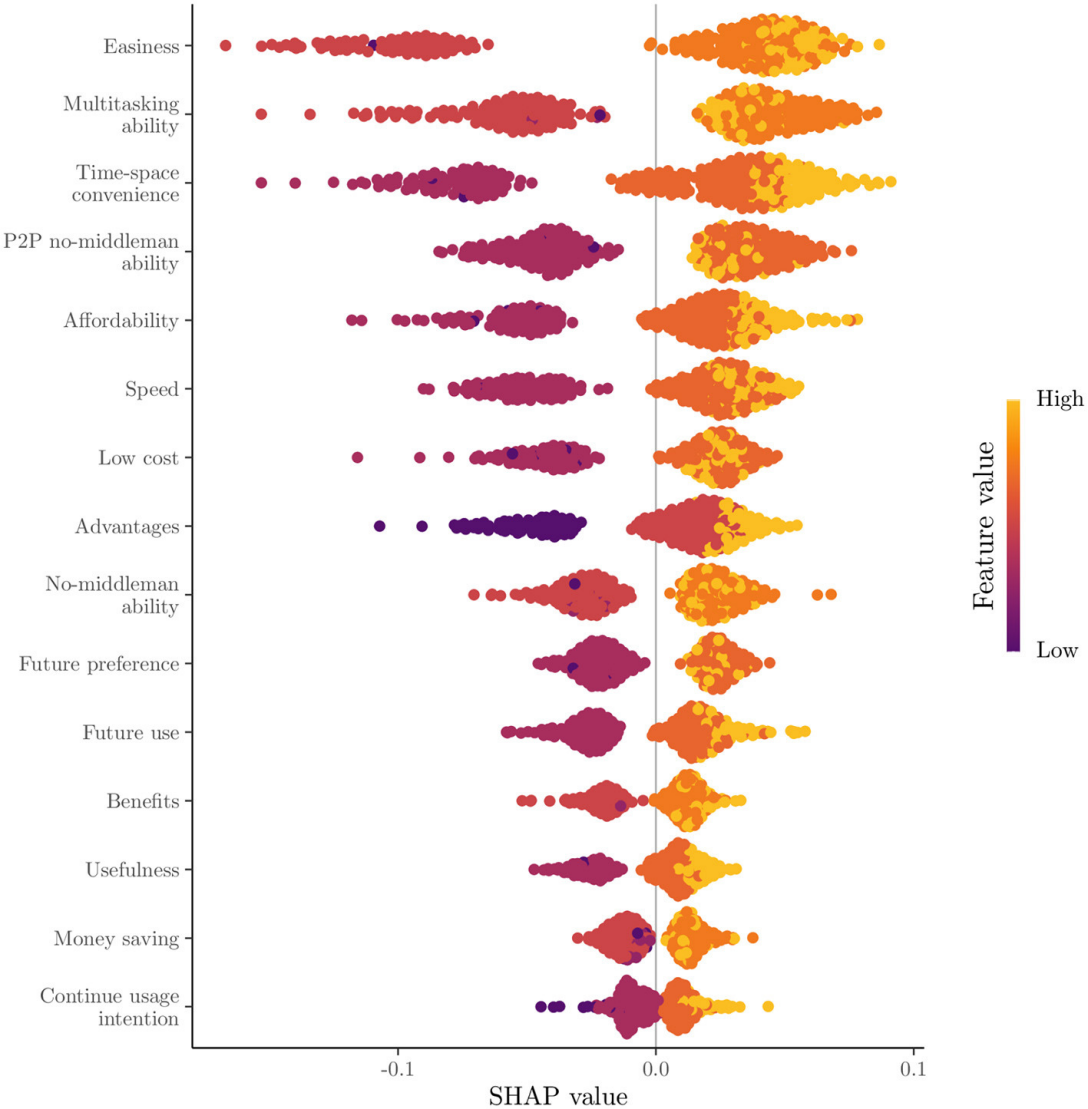
Global SHAP values bees warm plot.

**Figure 7:**
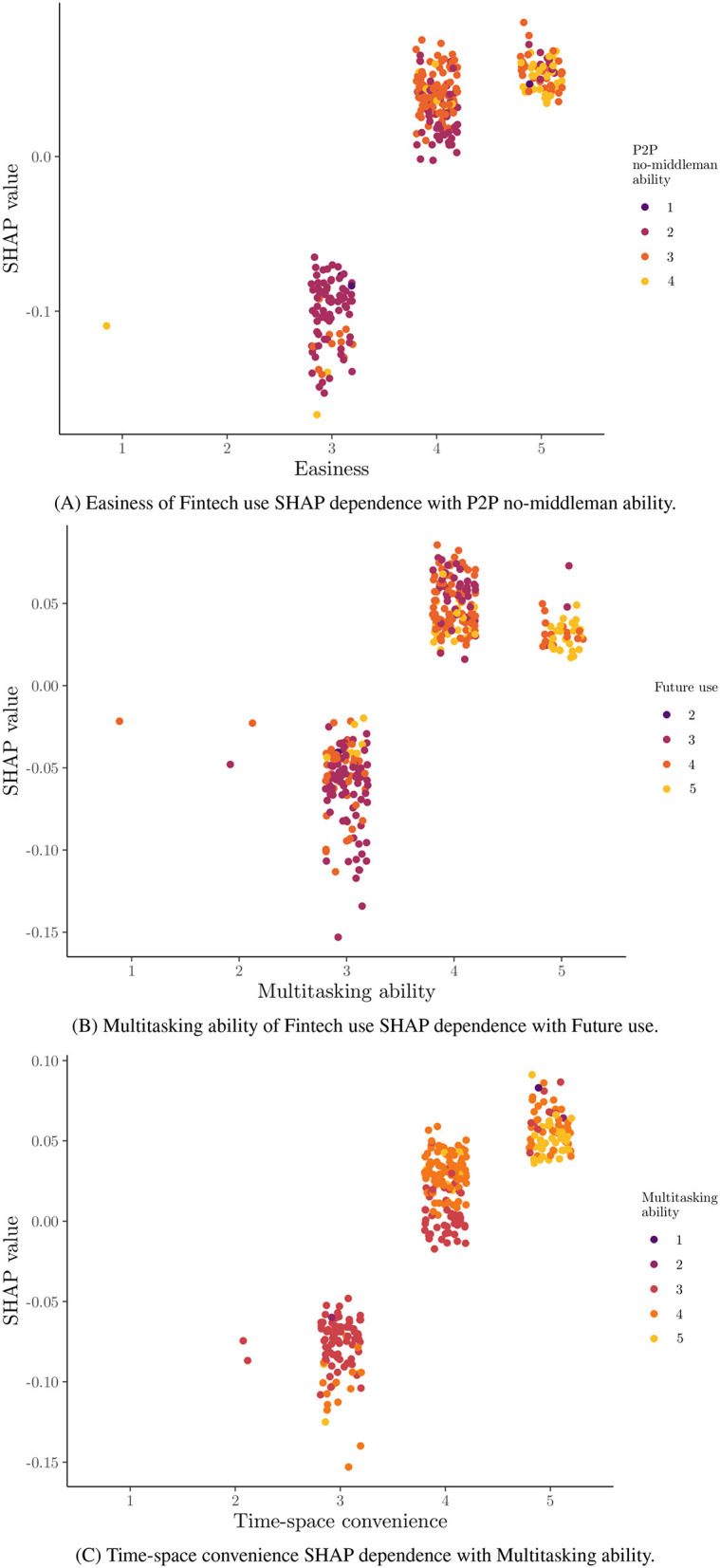
Jittered points SHAP dependence plots for three most important features. **(A)** Easiness of Fintech use SHAP dependence with peer-to-peer (P2P) no-middleman ability. **(B)** Multitasking ability of Fintech use SHAP dependence with future use. **(C)** Time-space convenience SHAP dependence with multitasking ability.

The feature importance is based on the mean absolute SHAP value per feature for the 15 most important features are presented in descending order in Figure 5. In this bar plot, it is clear that the three most important features, namely ease, multitasking ability, and time-space convenience, are about the benefits received from Fintech use compared to the traditional financial means. Despite these, the importance of features seems to decrease exponentially. In Figure 6, the global SHAP values for the same features are depicted by a bee swarm summary plot. In this illustration, each instance of the dataset is presented as a single point. All presented features have a positive contribution to the probability for a female student to be classified at Cluster 1, where high SHAP values are associated with high feature values. However, negative feature values will have a stronger negative impact on the probability for a female student to be classified at Cluster 1 for the most features, due to the left-skewed distribution of the SHAP values, where low feature values with negative contributions are located in the distribution's tail. Therefore, a positive association seems to be across the presented feature values and the prediction probability for Cluster 1. In Figure 7, the dependence plots of the three most important features are presented, given their SHAP values. Here, except for the positive association that was presented in Figure 6, there is also a positive relationship among illustrated features, because their high and low values are in accordance, and in most cases, only for low values of both, a negative contribution is shown; otherwise, it is positive.

## 4 Discussion

In this paper, a segmentation of female students based on their perceptions toward Fintech is attempted. As a result, the existence of two segments of female students was validated in the current study. The presence of two distinct clusters highlighted the diversity within the student population. The first segment consists of “Fintech-friendly” female students, and another one of “Fintech-skeptical.” The most prominent factors for positively classifying a woman in the “Fintech-friendly” cluster are the advantages of Fintech use over the traditional financial means. These factors align with the prior literature regarding the existence of Fintech-averse and Fintech-lover market segments (Jaiswal et al., 2023). Additionally, the factors affecting most and positively, the decision for classifying at the “Fintech-friendly” group is in relation to perceived benefits of Fintech usage. In contrast to previous studies, the demographic characteristics of the respondents were found to be irrelevant to this segmentation (Jaiswal et al., 2023; Khera et al., 2022).

Interestingly, demographic features of the respondents were not as influential as they were anticipated, deviating from the outcomes of earlier studies (Jaiswal et al., 2023; Alshari and Lokhande, 2022). However, these phenomena are in accordance with some recent literature findings and have been interpreted by the fact that no income or education barriers are faced by the sample (Alonso et al., 2023), where in this case the respondents are all on university level and so a basic education level is existent. As a result, women's behavior in this case of a developing country (International Monetary Fund, 2024) is driven mainly by social and psychological factors, opposed to cases of the least developing countries where demographic characteristics have greater influence (Alshari and Lokhande, 2022).

The significance of ease, multitasking ability, and time-space convenience was further highlighted as being the most significant predictors of cluster membership. Consequently, convenience and usability are paramount when choosing Fintech services over traditional financial methods (Sheppard and Vibert, 2019). The finding that higher feature values are associated with higher probabilities of adoption were reinforced by the positive contributions of these features to the probability of classification into the first cluster. Even though the argument that ease of use of technology is not linked to perceptions toward its usefulness (Sheppard and Vibert, 2019) is not directly checked, both of these factors are significant in this “Fintech-friendly” vs. “Fintech-sceptical” classification. While high feature values positively influence adoption, low values can have a disproportionately negative impact. By this way, female students are potentially deterred from using Fintech services (Sheppard and Vibert, 2019). This information is crucial for Fintech providers, as the need to not only enhance positive features but also mitigate negative ones were denoted.

The positive connection among the three most important features was verified by the dependence plots. In precision, with high values correlating with positive contributions to the classification probability for the first cluster. As a consequence, students who respect one positive component of Fintech are likely to value others as well, reinforcing the importance of a well-rounded approach to feature enhancement is suggested (Oyetade et al., 2020). In addition, higher ease in the use of Fintech services is positively related to P2P no-middleman ability, which means these women possibly do have some experience with Fintch tools, and so this is in correspondence with Alonso et al. (2023), where individuals with higher experience with such tools have a more positive view toward them.

Based on the aforementioned, researchers, marketing analysts, and policymakers could benefit from this work. For example, more focus on the deeper reasons for this two-class diversity could be done by researchers. Moreover, an increase in women adopting positive perceptions toward Fintech could be achieved by focusing more on possible benefits from financial technologies. Simplified interfaces, enhanced accessibility, and affordable financial solutions should be placed as a priority for enhancing adoption rates among young women. More attempts are needed for constructing Fintech supporting seamless transactions for promoting future intentions of Fintech use (Al Azizah et al., 2022). Common worries and misunderstandings around the use of technology in financial transactions should be addressed in campaigns by educating female students on utilizing Fintech services securely and efficiently. Thus, financial literacy should be a higher priority of all organizations, as long as it is considered to have a positive impact on Fintech adoption (Khera et al., 2022). The segmentation of the sample into two groups is evidence for customized consumer experience in Fintech marketing, approaching target groups of “Fintech-friendly” and “Fintech-sceptical” female students in a personalized way, according to their needs and concerns.

Despite the significant contributions of the current study, some limitations are faced. For example, only students are included in the sample. So, given the facts that the majority of university graduates in Indonesia are 24 years old or below (Welch and Aziz, 2022) and the majority of Fintech tool usage in Indonesia, like Fintech lending tools, is performed by the age group 19–35 (Abdurrahman et al., 2022); these results are not representative of the general female population. Additionally, the results of this study are related to a specific country (Indonesia). So, as long as consumer differences across cultures where multiple demographics, conceptions (Ares, 2018; Buil et al., 2012; Frost, 2020), languages, and responses to surveys are evidenced (Ares, 2018; Buil et al., 2012), hardening the generalization of the above results. For instance, Fintech tool usage is usually higher in the younger population (Frost, 2020). Moreover, other economic factors, such as unfulfilled financial needs and financial inclusion, low cost of Fintech tools, helpful regulation system, and macroeconomic conditions are different across countries, and thus producing varying perceptions toward Fintech services between them (Frost, 2020). Besides, a sample from a single university in Indonesia is not representative of the whole country population. The quality of the dataset might be affected by the self-reporting component of the dataset, as long as, especially in such surveys with psychological aspects toward an issue are dependent on the individuals' attitude. So the correspondents' opinion could be altered after some time (Gao et al., 2021). From a methodological aspect, the result outputs could be significantly affected by grouping the constructs into single features and by deploying additional classification and clustering methods. In addition, the outputs of the spectral clustering techniques are highly sensitive over the selection of hyperparameters, the choice of Laplacian matrix, and the characteristics of the input (Von Luxburg, 2007).

Extensions of the current study could be explored, including a more informative image of the global situation could be provided by a larger dataset with a greater variety of individual characteristics and background, like samples where multiple educational levels, social groups, and country origins and continents are combined. In other words, these findings could be further explored upon in future research by investigating additional factors that influence Fintech adoption across different demographic groups and cultural contexts (Jan et al., 2024; Liu et al., 2024). Besides, fruitful results could be revealed by surveying and testing based on the validity of other theoretical models, such as TAM and UTAUT. By focusing on these aspects, offerings could be better tailored by Fintech means providers to meet the needs and demand of this demographic, thereby increasing user satisfaction and adoption rates. Employment of other statistical and machine learning methods and ensembles of them for clustering, such as GMM and Self-Organizing Maps (SOMs) for mixed data, classification, including Gradient Boosting Machines (GBM), Neural Networks (NN), or Support Vector Machines (SVM), and interpretation, like Accumulated Local Effects (ALE) and Partial Dependence (PD) methods.

Longitudinal study design for assessment of the evolution of the preferences and perceptions of Fintech services through time could be involved as another extension as well. Insights into whether the identified key features, such as ease, multitasking ability, and time-space convenience, remain constant in importance or change as students gain more experience with Fintech means could be provided. The long-run effects of Fintech tool usage on financial behavior and decision-making among students could also be examined. Despite the fact that longitudinal studies regarding consumers' behavior are performed in recent literature (Yang and Zhang, 2022), it is not concentrated mainly on the gender gap and specialized on women's aspects (Fox-Robertson and Wójcik, 2024). The combination of such studies with qualitative is depicted as quite insightful, according to the recent literature, accompanied by raised awareness on the regarding results' generalization (Fox-Robertson and Wójcik, 2024).

The results of this study may be expanded upon in further research, enhancing the efficacy of Fintech services in meeting the requirements of various female students and the general population demographics and providing a more thorough understanding of the variables driving Fintech adoption.

## 5 Conclusions

The analysis conducted in this study provided valuable insights into the factors that influence female Indonesian university students' preferences for utilizing Fintech services. Six features, primarily demographic details, were identified and removed from the analysis as irrelevant with the influence perceptions of Fintech services by using the USFSM method. Thereafter, the presence of two distinct clusters in the dataset were revealed by the eigengap heuristic and Fiedler vector partitioning, in the context of spectral clustering. A total of 189 female students were contained in the first cluster and 126 in the second one. Moreover, higher values for features such as ease, time-space convenience, and affordability were generally preferred by female students in the first cluster compared to those in the second one. The prominent importance of three key features, specifically ease, multitasking ability, and time-space convenience were highlighted by the SHAP value analysis of the Random Forest classification model for these two clusters. All presented features have a positive contribution to the probability of a female student being classified into the first cluster, with higher feature values leading to higher prediction probabilities for the first cluster. A significant impact on the classification probability was observed for low feature values with negative contributions attributed to the left-skewed distribution of SHAP values. The dependence plots of the three most important features confirmed a positive relationship between them the relationship was high. The values were correlated with positive contributions to the classification probability for the first cluster. There is accordance of these results with the existing literature, but generalizations limitations are faced about the used sample, its demographics, and methods. Implications about usability, convenience, Fintech education, and personalized experiences could be beneficial practices for Fintech stakeholders.

## Data Availability

Publicly available datasets were analyzed in this study. This data can be found here: https://data.mendeley.com/datasets/6ncwmyx6y4/3.

## Footnotes

^1^In this study, the term “Fintech” is used both in the sense of financial services and in the perception toward standard and innovative financial products issued via technological tools and to technological application generally used in Fintech.
